# Supplementation with Nicotinamide Riboside Reduces Brain Inflammation and Improves Cognitive Function in Diabetic Mice

**DOI:** 10.3390/ijms20174196

**Published:** 2019-08-27

**Authors:** Hee Jae Lee, Soo Jin Yang

**Affiliations:** Department of Food and Nutrition, Seoul Women’s University, Seoul 01797, Korea

**Keywords:** amyloidogenesis, cognitive impairment, neuroinflammation, nicotinamide riboside

## Abstract

The purpose of this study is to investigate whether nicotinamide riboside (NR) can improve inflammation and cognitive function in diabetic mice. ICR male mice were fed for 14 weeks with either high-fat chow diet (HF, 60% kcal fat) or standard chow diet (CON, 10% kcal fat). HF, streptozotocin, and nicotinamide were used to induce hyperglycemia. NR or vehicle was delivered via stomach gavage for six weeks. Oral glucose tolerance test, Y-maze test, and nest construction test were conducted before and after the NR treatment period. NR treatment induced down-regulation of NLRP3, ASC, and caspase-1. NR reduced IL-1 expression significantly by 50% in whole brains of hyperglycemic mice. Other inflammatory markers including TNF-α and IL-6 were also attenuated by NR. Brain expression of amyloid-β precursor protein and presenilin 1 were reduced by NR. In addition, NR induced significant reduction of amyloid-β in whole brains of diabetic mice. NR treatment restored hyperglycemia-induced increases in brain karyopyknosis to the levels of controls. Nest construction test showed that NR improved hippocampus functions. Spatial recognition memory and locomotor activity were also improved by NR supplementation. These findings suggest that NR may be useful for treating cognitive impairment by inhibiting amyloidogenesis and neuroinflammation.

## 1. Introduction

Alzheimer’s disease (AD) is the most common form of dementia. It impairs cognitive function and performance of daily life. Aging, genetic factors, and environmental factors including diet, alcohol, and air pollutions contribute to the development of AD [1]. Neuropathologically, AD is characterized by excessive formation of amyloid-beta plaque and neurofibrillary tangle [2,3]. Amyloid-beta is generated by proteolytic cleavage of amyloid-beta precursor protein (APP) with beta- and gamma-secretases [4]. Presenilin (PS) 1, one of core proteins in gamma-secretase complex core, is considered a regulatory component for amyloid beta generation [5,6]. Monomers of amyloid-beta aggregate to form its multimers, subsequently forming amyloid-beta plaque. These formed amyloid beta plaques can disrupt neuronal structure/integrity on deposited areas and lead to neuronal dysfunction [7]. Tau is a microtubule-associated protein. It is abnormally phosphorylated and aggregated, forming neurofibrillary tangle in AD [2,8].

Hyperglycemia increases risk of cognitive impairment and developing dementia [9,10,11] which is also termed as “diabetes-related dementia” [12,13,14]. Epidemiological study suggests that high concentrations of blood glucose increase risk of dementia, even in those without diabetes [15]. Several experimental studies using animal models have demonstrated that hyperglycemia-induced brain insulin resistance causes AD-like alterations [16,17]. In addition to brain insulin resistance, amyloidogenesis, neuroinflammation, oxidative stress, and autophagy may contribute to the development of diabetes-related dementia [3,13,18,19,20,21,22].

In general, amyloid beta monomers are not toxic. However, once they assemble and aggregate to form its multimers, they become more insoluble and neuritic plaques [22]. Amyloid-beta aggregation stimulates excessive production of pro-inflammatory cytokines including interleukin (IL)-1 beta and tumor necrosis factor (TNF)-alpha in monocytes and microglia [23,24,25]. Excessive levels of systemic and neuronal inflammation aggravate the progression of AD [22,26]. Amyloid-β increases levels of IL-1β and TNF-alpha and activates the nucleotide binding and oligomerization domains-like receptor family, pyrin domain containing 3 (NLRP3) inflammasome in mouse microglia [27]. Genetic deficiency of *NLRP3* in *APP*/*PS1* transgenic mice protects against spatial memory impairment, loss of hippocampal synaptic plasticity, and behavioral disturbances [28]. In addition, hyperglycemic condition may activate inflammatory responses [29]. Impaired glucose hemostasis and neuronal insulin resistance might trigger amyloid-β aggregation and tau hyperphosphorylation [29].

Nicotinamide riboside (NR) is a natural NAD^+^ precursor and one of vitamin B_3_ [30]. Excitotoxicity-induced axonal degeneration occurring in chronic neurodegenerative diseases can be protected by NR injection in mice [31]. NR treatment can improve cognitive function through up-regulation of proliferator-activated receptor-γ coactivator 1α-regulated degradation of β-secretase 1 in Tg2576 transgenic mice [32]. NR supplementation prevents the development of diabetic peripheral neuropathy involving sensory and motor neurons in a rodent model of type 2 diabetes induced by high fat (HF) diet [33]. However, the possible effect of NR on hyperglycemia-induced dementia and its underlying mechanisms remain unclear. Therefore, the aim of this study is to investigate whether hyperglycemia can induce impairment of cognitive function and whether NR can attenuate hyperglycemia-induced cognitive impairment in a rodent model of type 2 diabetes induced by HF diet feeding and streptozotocin (STZ)-nicotinamide injection. We also focused on amyloidogenesis and neuroinflammation as potential regulatory mechanisms for hyperglycemia-induced cognitive impairment.

## 2. Results

### 2.1. Effects of NR on Body Weight, Brain Weight, Food Intake, and 2 h Oral Glucose Tolerance Test Area under the Curve (OGTT AUC)

The weights of whole brain were significantly increased by 6 weeks of NR treatment, while there were no significant differences in body weight among groups (Table 1). HF had no effect on food intake. NR treatment did not affect food intake of HF-fed mice either. The fasting blood glucose concentrations and 2 h OGTT AUC were significantly increased by HF diet and STZ injection. Such increases were not normalized by NR administration.

### 2.2. NR Reduces Levels of Inflammatory Markers in Whole Brains of Mice

**Figure 1:**
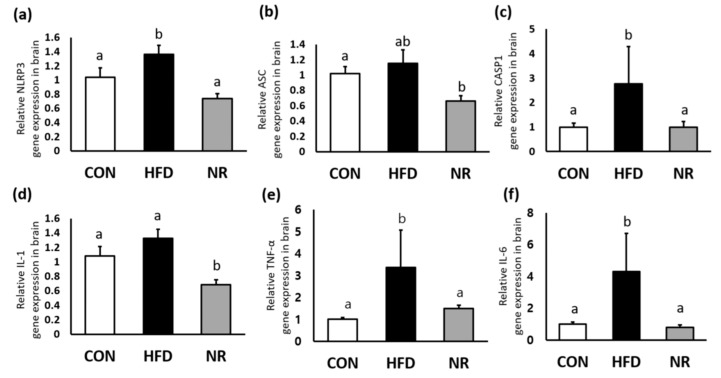
The effects of nicotinamide riboside (NR) on the nucleotide binding and oligomerization domain-like receptor family, pyrin domain containing 3 (NLRP3) inflammasome components and inflammatory markers in whole brains of mice. Relative gene expression of (**a**) *NLRP3*, (**b**) *apoptosis-associated speck-like protein containing a caspase recruitment domain* (*ASC*), (**c**) *caspase 1* (*CASP1*), (**d**) *interleukin* (*IL)-1*, (**e**) *tumor necrosis factor (TNF)-α*, and (**f**) *IL-6*. Values represent the mean ± SEM (*n* = 5 per group). Values with different letters in the same variable are significantly different (*p* < 0.05). CON, control; HFD, high-fat diet.

HF increased gene expression levels of *NLRP3* and its component *caspase-1* (*CASP1*) in the brain, whereas NR treatment reduced their expression levels similar to the those of control mice (Figure 1a,c). In addition, NR administration down-regulated gene expression levels of *IL-1*, *TNF-alpha*, and *IL-6* in the brain (Figure 1d–f).

### 2.3. NR Supplementation Reduces Amyloid-β Concentrations in Whole Brains of Mice

**Figure 2:**
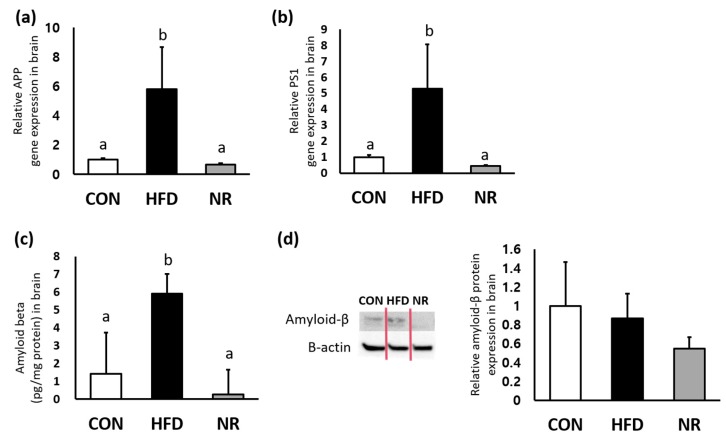
The effects of nicotinamide riboside (NR) on amyloid-beta precursor protein (APP), presenilin1 (PS1), and amyloid-β in the whole brain of mice. Relative gene expression of (**a**) *APP* and (**b**) *PS1*. (**c**) Amyloid beta concentrations in brain were analyzed by ELISA. (**d**) Representative Western blot for amyloid beta and beta-actin, and their respective quantification in brain. Values represent the mean ± SEM (*n* = 5 per group). Values with different letters in the same variable are significantly different (*p* < 0.05). CON, control; HFD, high-fat diet.

To examine whether NR can affect levels of amyloid-β, enzyme-linked immunosorbent assay (ELISA) and Western blot were performed for measuring amyloid-β levels in whole brains. In addition, gene expression levels of *APP* and *PS1* were analyzed. Gene expression levels of *APP* and *PS1* were significantly up-regulated by HF feeding, whereas NR treatment down-regulated *APP* and *PS1* expressions in whole brains (88% and 91%, respectively) (Figure 2a,b). As shown in Figure 2c, HF feeding increased amyloid-β concentration in the brain, whereas such increase was significantly reduced (95% decreases) by 6 weeks of NR treatment. Western blot analysis showed NR-mediated decreasing tendency of amyloid β in the brain lysates of HF diet-fed mice, although such decrease was not statistically significant (Figure 2d).

### 2.4. NR-Mediated Morphological Changes in Brain Tissues of HF Diet-Fed Mice

**Figure 3:**
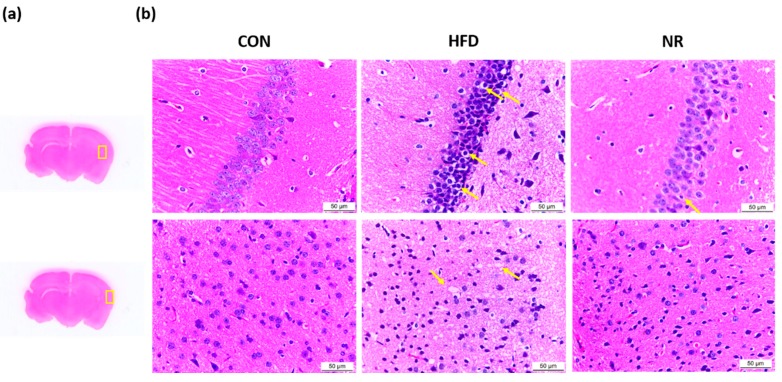
Representative photographs of hematoxylin and eosin (H and E)-stained brain sections. (**a**) The areas examined in the brain were marked with yellow boxes. (**b**) H and E-stained brain sections. Original magnification 400×. Yellow arrows indicate karyopyknosis of cells. CON, control; HFD, high-fat diet; NR, nicotinamide riboside.

Morphological changes were assessed using hematoxylin and eosin (H and E) staining (Figure 3). Areas examined in the brain are shown in Figure 3a. Brain H and E staining showed that brain sections of untreated HFD mice had disorderly arrangement with loosened cytoplasm and karyopyknosis (Figure 3b). Karyopyknosis in the brain was observed in aluminum-induced brain damage or diabetic brain of rats [34,35]. The administration of NR decreased the extents of karyopyknosis (Figure 3b) in brain sections. Also, nerve cells in NR-treated mice were more closely arranged compared to those in HFD mice.

### 2.5. Nest Construction Test and Y-maze Test

**Figure 4:**
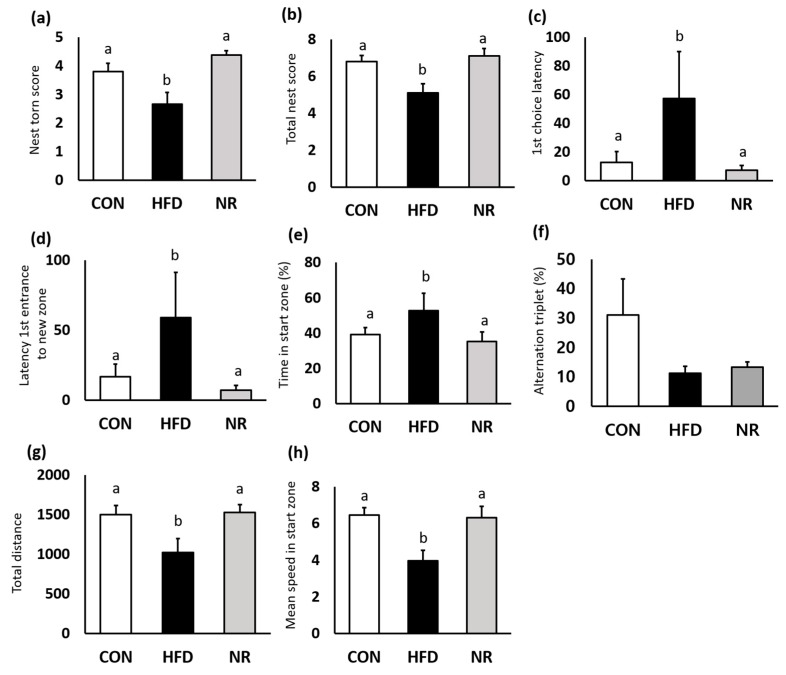
The effects of nicotinamide riboside (NR) on behavior tests. (**a**) Nest torn score and (**b**) total nest score were evaluated from nest construction test. (**c**) First choice latency, (**d**) latency first entrance to new zone, (**e**) time in start zone (%), (**f**) alternation triplet (%), (**g**) total distance, and (**h**) mean speed in start zone are analyzed from the Y-maze test. Values represent the mean ± SEM. Values with different letters in the same variable are significantly different (*p* < 0.05). CON, control; HFD, high-fat diet.

Nest construction test was performed to assess the hippocampus function. HFD mice barely touched nest materials, whereas nestlets were most shredded by NR-treated mice (Figure 4a). Six weeks of NR treatment significantly improved the total nest score by 27% compared with HFD mice (Figure 4b).

Y-maze test was performed to evaluate spatial working and recognition memory. HFD mice displayed impaired memory and locomotor activity. First-choice latency and latency of the first entrance to new zone indicate indecision and response time. NR treatment significantly improved these factors (Figure 4c,d). Although NR treatment did not affect alternation triplet, NR-treated mice spent significantly less time in start arm (Figure 4e,f). In addition, NR treatment increased locomotor activity (Figure 4g,h). Total distance during the test and mean speed in start zone were significantly increased in the NR-treated mice.

## 3. Discussion

In the present study, we demonstrated effects of NR on amyloidogenesis and inflammation in whole brains of diabetic mice. Administration of NR reduced amyloid-β and several inflammatory markers including NLRP3, CASP1, IL-1, TNF-alpha, and IL-6 in brains of HF diet-fed mice. NR treatment ameliorated disorganized structure and excessive karyopyknosis in brains. These attenuations induced improvements in hippocampal function and spatial working/recognition memory.

A rodent model of diabetes exhibits AD-related alterations such as increases in APP, amyloid-β, and phosphorylated tau [16,36,37]. These changes are associated with cognitive impairment. In the present study, male ICR mice were fed with HF diet for 14 weeks and injected with STZ and nicotinamide. HF diet feeding and STZ injection established a rodent model of diabetes with AD-like alterations. Impairments in cognitive function were evidenced by decreases in total nest construction score as well as deteriorations in spatial working/recognition memory during the Y-maze test. It has been suggested that diabetes might cause AD [29,38] which is considered as a complication of diabetes [39]. Hyperglycemia impairs insulin signaling that can affect the synthesis and degradation of amyloid-β. Amyloid-β is produced by proteolytic cleavage of APP with enzymatic action of β-secretase and γ-secretase [40]. Unexpectedly, NR treatment did not improve glycemic control during OGTT in the present study. In mice, NR supplementation prevented the development of diabetic neuropathy while improving glucose tolerance in a rodent model of type 2 diabetes [33]. However, administration of NR for 12 weeks had no effects on insulin sensitivity or glucose control in insulin-resistant obese individuals [41]. Differences in treatment duration and severity of diabetes might have caused unexpected results. However, NR treatment improved hippocampus function and spatial working/recognition memory by reducing amyloid-β deposit in whole brains of diabetic mice. These findings suggest that NR might attenuate the pathophysiological process of hyperglycemia-induced accumulation of amyloid-β. NR-mediated decreases in amyloid-β have also been reported in another animal model of AD, consistent with our findings [32].

Inflammatory reaction in the brain is closely related to cognitive impairment [42,43,44]. HF diet feeding affects brain homeostasis and metabolic pathways. HF feeding for 16 weeks altered molecular targets of insulin signaling, innate immune system, and inflammation in hippocampus of rats, which are related to the aggravation of anxiety and depression [45]. Also, 14 weeks of HF diet feeding induced brain insulin resistance and cognitive impairments with excessive responses of pro-inflammatory pathways in whole brain lysate of mice [46]. STZ-induced hyperglycemic condition increased the levels of IL-6, TNF-α, and cyclooxygenase-2 in brain tissues of rats [44]. These increases in inflammatory cytokines are involved in the pathogenesis of diabetes-related cognitive dysfunction in diabetic rats [44]. In the current study, we found that NR treatment decreased proinflammatory cytokines IL-1, TNF-α, and IL-6 in the brains of diabetic mice. In addition, NLRP3 was reduced by NR treatment. NOD-like receptors (NLRs) represent one of amyloid-β sensing systems [47]. In AD, amyloid-β oligomers and fibrils can trigger NLRP3 activation in microglia [27]. NLRPs activate apoptosis-associated speck-like protein containing a caspase recruitment domain (ASC) that induces caspase activation as well as maturation of pro-inflammatory mediators such as IL-1β and IL-18 [48]. Ablation of NLRP3 enhances insulin signaling in a mouse model of obesity [49]. *NLRP3* gene deletion had protective effects on cognitive impairment in *APP/PS1* transgenic mice [28]. In the present study, NR treatment improved hippocampus function and memory while reducing inflammatory markers including NLRP3. These results suggest that cognitive impairment induced by diabetes may be associated with NLRP3 signaling.

Brain morphological impairments have been observed in AD patients and animal models of AD [35,50,51]. Karyopyknosis on hippocampus has been observed in chronic diabetic rats [34,51]. In our study, administration of NR decreased karyopyknosis. Also, brain nerve cells in NR-treated mice were closely arranged similar to those of normal condition. Collectively, these results suggest that NR treatment can ameliorate morphological impairments induced by hyperglycemia.

In summary, NR treatment reduced neuroinflammation and amyloidogenesis in the whole brain. In addition, impaired cognitive function as shown by low nest construction score and deficits in spatial working and recognition memory can be attenuated by 6 weeks of NR treatment. Findings of the current study suggest that NR supplementation is beneficial for maintaining proper cognitive functioning and preventing the development of hyperglycemia-related dementia by inhibiting neuroinflammation and amyloidogenesis.

## 4. Materials and Methods

### 4.1. Animals

Male ICR mice (6 weeks of age) were obtained from Raon Bio (Yongin, Gyeonggi-do, Korea). Mice were allowed access freely to diet and water. Animal housing rooms were maintained at a constant room temperature in a 12-h light (7:00 A.M.)/dark (7:00 P.M.) cycle. After one week of adaption to the laboratory conditions, ICR mice were placed with either standard chow diet (control; 10% kcal fat, D12450B, Research diet, New Brunswick, NJ, USA) or high-fat chow diet (HF; 60% kcal fat, D12492). Nicotinamide was injected to the mice assigned to HF groups at treatment week 4 to protect them from severe pancreatic beta cell damage induced by STZ [52]. After 15 min from nicotinamide injection, STZ (100 mg/kg, dissolved in 0.1 M citric acid buffer (pH 4.4)) was injected to the mice. STZ-nicotinamide injection is considered as a standard way to induce type 2 diabetes [52]. STZ and nicotinamide were purchased from Sigma-Aldrich (Sigma-Aldrich, St Louis, MO, USA). Hyperglycemia was confirmed with a fasting glucose level at treatment week 8. To assess fasting glucose levels, mice were fasted for 3 h before the test. The blood samples were taken from the tail of the mice. Glucocard 01 Sensor (Arkray, Seoul, Korea) was used to measure glucose concentrations. The fasting glucose levels were 102 ± 11 mg/dL for control group and 425 ± 29 mg/dL for all HF groups (CON vs. HF groups, *p* < 0.001). The mice fed with HF diet were randomly divided into two groups; (1) HF diet group (HFD) and (2) HF + NR (400 mg/kg/day) group (*n* = 8 per group). Control and HFD mice were administered with saline, and NR group mice were treated with NR by oral gavage daily for additional 6 weeks. Mice of HFD and HF +NR groups were fed with HFD for total 14 weeks. Body weights and food intake were monitored weekly. After 6 weeks of NR treatment, mice were sacrificed. This study protocol conformed to the specifications outlined in the National Institutes of Health Guiding Principles for the Care and Use of Laboratory Animals, and was approved by the Institutional Animal Care and Use Committee of the Seoul Women’s University (Approved protocol number: SWU IACUC-2017A-4, approval date: 3 August 2017).

### 4.2. OGTT

After an overnight fasting for 16 h, OGTT was performed for all experimental mice. Blood samples were drawn initially and then the animals were fed with glucose solution (2 g/kg body weight) via stomach gavage. At 15, 30, 60, 90, and 120 min after the glucose challenge, blood samples were obtained and used for glucose measurement using Glucocard 01 Sensor (Arkray). AUC was calculated based on the glucose concentrations obtained during OGTT.

### 4.3. Nest Construction Test

The nest construction test was performed before Y-maze test. White cotton pads (50 × 50 mm) were placed into individual cages with mice. The test started from 7:00 PM. After 24 h, each pad and nest shape were weighted and recorded. The experimental procedures and scoring system were conducted according to the protocol previously published [53,54]. The total nest scores evaluate the shape of nest and the amount of material unused.

### 4.4. Y-maze Test

To assess whether NR treatment affects spatial working and recognition memory, behavioral tests were conducted at before and after 6 weeks of treatment. The Y-maze consists of three arms (white plastic walls and black floor). The test was conducted with two sessions. On the first session, each mouse was allowed to move freely for 5 min to test spatial working memory. The second session was conducted for testing spatial recognition memory. On the second session, one arm was blocked while the mouse explored the other two arms. After 15 min break time, three arms were opened to explore. The movements of mice were recorded and analyzed with a video-computerized tracking system (SMART, Panlab Harvard Apparatus, Holliston, MA, USA).

### 4.5. Histological Study

The H and E staining was used to assess the morphological changes. Brain tissues (*n* = 3 per group) were fixed with 4% paraformaldehyde. Fixed brain tissues were embedded in paraffin, sectioned in 5 μm thickness, and stained with H and E. H and E-stained sections were observed and imaged using a LEICA DM750 microscope with an integrated ICC50 camera (LEICA Microsystems, Wetzlar, Germany).

### 4.6. RNA Extraction and Gene Expression Determination

Total RNA from the whole brain was isolated using Invitrogen PureLink RNA Mini Kit (Thermo Fisher Scientific, Waltham, MA, USA). Applied Biosystems High Capacity cDNA Reverse Transcription (RT) Kit (Thermo Fisher Scientific) was used for cDNA synthesis. The total reaction volume for RT was 20 μL, including random primers, deoxynucleoside triphosphate mix, reverse transcriptase, RT buffer, and 5 µg total RNA with RNase free ddH_2_O. The RT conditions were 25 °C for 10 min, 37 °C for 120 min, 85 °C for 5 min, and 4 °C. The real-time PCR was performed using Applied Biosystems Power SYBR Green Master mix (Thermo Fisher Scientific) on a StepOne Plus Real-Time PCR System (Applied Biosystems). 18S was used as the control for sample normalization. Relative gene expression was calculated with ΔΔ*C*t method.

### 4.7. Western Blot Analysis

Whole brain (100 mg) was added with 1 mL of cold RIPA lysis buffer containing protease inhibitor and phosphatase inhibitor. The protein was isolated by syringe lysis and subsequent centrifugation at 10,000× *g* for 20 min at 4 °C, and the supernatant was applied for Western blot analysis. The protein concentration was determined using the Bradford method. A total of 50 µg protein was separated on 4–20% of sodium dodecyl sulfate polyacrylamide gel electrophoresis. Protein was transferred onto a polyvinylidene difluoride membrane. The membrane was blocked for 1 h using tris-buffered saline with Tween 20 containing 5% fat-free milk. The blot was incubated overnight at 4 °C with primary antibodies for Aβ (Abcam, Cambridge, UK) detecting amyloid β protein fragment 1–42 and β-actin (Cell Signaling Technology, Danvers, MA, USA). Then, the membrane was incubated for 1 h at room temperature with a secondary antibody conjugated to horseradish peroxidase for 1 h at room temperature. The bands were visualized with ECL reagents and detected by the chemi-luminescent imaging analyzer. Relative band intensities of Western blots were calculated with Image J software. The protein level of amyloid-β was normalized with the corresponding β-actin protein level.

### 4.8. ELISA

Amyloid-β concentrations in whole brain tissues were measured with commercial Amyloid-β ELISA kit to detect amyloid β protein fragment 1-42 (Invitrogen). Whole brain homogenates from each group were prepared with lysis buffer, followed by centrifugation at 10,000× *g* for 20 min under 4 °C. The supernatant was collected. A total of 50 µL of standards and samples were added to wells in the plate. Amyloid-β detection antibody solutions were added to each well containing standard or sample. Then, the plate was incubated for 3 h at room temperature. After the incubation time, each well was washed four times with wash buffer. And, anti-rabbit IgG HRP antibody was added to each well and incubated for 30 min at room temperature. Each well was washed, and stabilized chromogen solution was added to the wells. The stop solution was added to stop the reaction within 30 min. The plate was read at absorbance 450 nm with the microplate reader. The level of amyloid-β was normalized to protein concentration.

### 4.9. Statistical Analysis

Values were presented as means ± SEM and analyzed on SPSS statistics 24 (IBM Corp, Armonk, NY, USA). Among groups, differences were evaluated by a one-way analysis of variance (ANOVA) and Tukey’s post-hoc analysis. Statistical significance was considered with *p* < 0.05.

## Figures and Tables

**Table 1:** The effects of nicotinamide riboside (NR) treatment on body weight (BW), brain weight, food intake, and 2 h oral glucose tolerance test area under the curve (OGTT AUC).

	CON	HFD	NR
BW (g)	44.69 ± 1.31	45.90 ± 2.84	44.05 ± 2.61
Brain weight (g)	0.55 ± 0.01 ^ab^	0.52 ± 0.01 ^a^	0.59 ± 0.01 ^b^
Brain weight (% BW)	1.27 ± 0.05 ^ab^	1.14 ± 0.05 ^a^	1.36 ± 0.10 ^b^
Food intake (g/day)	3.82 ± 0.15 ^a^	3.16 ± 0.11 ^ab^	2.28 ± 0.40 ^b^
Fasting blood glucose (mg/dL)	123 ± 6 ^a^	389 ± 41 ^b^	383 ± 50 ^b^
2 h OGTT AUC	35099 ± 2538 ^a^	65091 ± 2619 ^b^	68648 ± 861 ^b^

Data are expressed as mean ± SEM (*n* = 8 per group). Different letters within a variable are significantly different at *p* < 0.05. AUC, area under the curve; CON, control; HFD, high-fat diet; OGTT, oral glucose tolerance test.

## Abbreviations

AD	Alzheimer’s disease
APP	amyloid-beta precursor protein
ASC	apoptosis-associated speck-like protein containing a caspase recruitment domain
NLRP3	nucleotide binding and oligomerization domains-like receptor family: pyrin domain containing 3
NR	nicotinamide riboside
PS1	presenilin1
